# Autoimmune Type 1 Diabetes: An Early Approach Appraisal for Spain by the AGORA Diabetes Collaborative Group

**DOI:** 10.3390/jcm14020418

**Published:** 2025-01-10

**Authors:** Fernando Gómez-Peralta, Pedro J. Pinés-Corrales, Estefanía Santos, Martín Cuesta, Olga González-Albarrán, Sharona Azriel, Luis Castaño, Chantal Mathieu, on behalf of the AGORA Diabetes Collaborative Group

**Affiliations:** 1 Endocrinology and Nutrition Unit, Hospital General de Segovia, 40002 Segovia, Spain; 2 Endocrinology and Nutrition Service, Complejo Hospitalario Universitario de Albacete, 02008 Albacete, Spain; ppines77@hotmail.com; 3 Endocrinology and Nutrition Service, Complejo Hospitalario de Burgos, 09006 Burgos, Spain; esantos@alumni.unav.es; 4 Endocrinology and Nutrition Service, Hospital Clínico San Carlos, 28040 Madrid, Spain; cuestamartintutor@gmail.com; 5 Endocrinology and Nutrition Service, Hospital Gregorio Marañón, 28007 Madrid, Spain; olgagonzalezalbarran@gmail.com; 6 Endocrinology and Nutrition Service, Hospital Universitario Infanta Sofía, 28702 San Sebastián De Los Reyes, Spain; sharona.azriel@gmail.com; 7 Biobizkaia Health Research Institute, Pediatric Endocrinology Department, Cruces University Hospital, UPU/EHU, Centro de Investigación Biomédica en Red de Enfermedades Raras (CIBERER), Centro de Investigación Biomédica en Red de Diabetes y Enfermedades Metabólicas Asociadas (CIBERDEM), Endo-ERN, 48903 Barakaldo, Spain; luisantonio.castanogonzalez@osakidetza.eus; 8 Clinical and Experimental Endocrinology, Department of Chronic Diseases and Metabolism (CHROMETA), KU Leuven, 3000 Leuven, Belgium; chantal.mathieu@uzleuven.be

**Keywords:** autoimmune diabetes, type 1 diabetes, pathogenesis, disease stages, screening, prevention, pediatric population, Spain

## Abstract

Type 1 diabetes (T1D) is an autoimmune disorder characterized by the destruction of insulin-producing pancreatic beta-cells, leading to lifelong insulin dependence. This review explores the current understanding of T1D pathogenesis, clinical progression, and emerging therapeutic approaches. We examined the complex interplay between genetic predisposition and environmental factors that could trigger the autoimmune response as well as the immunological mechanisms involved in beta-cell destruction. The clinical phases of T1D are discussed from the preclinical stage through diagnosis and long-term management, highlighting the importance of early detection and intervention. Recent advancements in treatment strategies are presented, including immunomodulatory therapies and potential cell-based treatments aimed at preserving or restoring beta-cell function. Additionally, this review critically evaluates the feasibility and potential benefits of implementing a population-wide screening program for T1D in Spain. The epidemiological, economic, and ethical implications of such an initiative were considered by the national expert panel, focusing on the potential of early diagnosis to improve clinical outcomes in the face of the challenges of large-scale implementation. This comprehensive analysis aims to provide healthcare professionals, researchers, and policymakers with valuable insights into the current landscape of T1D management and prospects for enhanced prevention and treatment strategies in the Spanish context.

## 1. Introduction: Type 1 Diabetes, an Autoimmune Disease

Type 1 diabetes (T1D) is a chronic autoimmune condition characterized by immune-mediated destruction of pancreatic beta-cells, leading to progressive loss of insulin production that eventually requires lifelong insulin therapy for survival [1]. The detection of islet-reactive autoantibodies provided the first strong evidence in favor of an autoimmune-mediated pathogenesis of T1D [2]. Several autoantigens have been defined in recent decades (InsB: 9–23, GAD65, IA-2, GRP78, etc.) [3]. The last and most robust proof of the autoimmune nature of T1D was the delay of disease onset resulting from immunomodulatory agents [4]. However, what leads to the loss of tolerance and autoimmune attack in T1D remains to be answered to fully understand the pathogenesis of the disease.

It is known that the autoimmune nature of T1D involves a complex interplay between genetic predisposition and environmental triggers [5]. There is a strong genetic component, with certain alleles of the human leukocyte antigen (HLA) complex being major risk factors [6]. However, not all individuals with these genetic markers develop T1D, indicating that environmental factors also play a critical role. Maternal and intrauterine conditions, mode of delivery, viral infections, gut microbiome composition, antibiotic exposure, and/or dietary habits are thought to contribute to the initiation/progression of autoimmunity [7,8].

**Figure 1:**
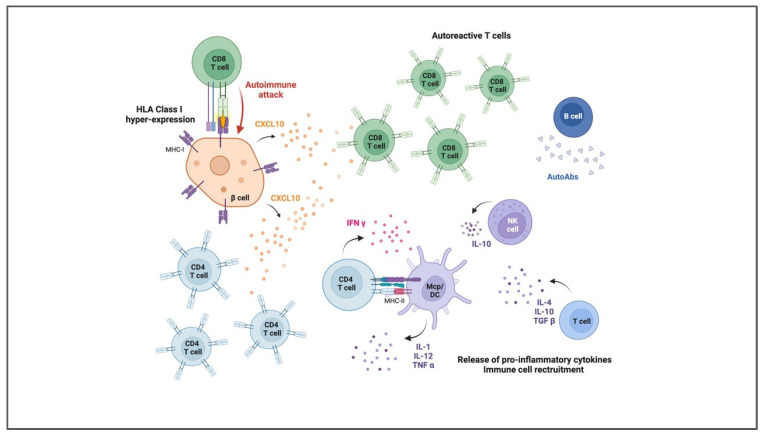
How are beta-cells destroyed in T1D?

Autoreactive T cells primarily mediate autoimmune attacks on beta-cells. CD8+ cytotoxic T lymphocytes directly attack beta-cells, recognizing specific antigens presented by class I HLA molecules on the surface of beta-cells (Figure 1). Additionally, CD4^+^ helper T cells contribute to the destruction by producing proinflammatory cytokines that recruit other immune cells and sustain the inflammatory response within islets. The infiltration of pancreatic islets by immune cells, a process known as insulitis, is a hallmark of early T1D, which leads to progressive beta-cell destruction [9]. B-cells also participate in the autoimmune response by producing autoantibodies against beta-cell antigens, such as insulin, glutamic acid decarboxylase (GAD), and tyrosine phosphatase IA-2. While these autoantibodies are hallmark features of T1D and serve as key biomarkers for predicting disease onset, their direct role in beta-cell destruction has not been clearly established. They may facilitate the presentation of autoantigens to T cells or activate complement pathways that contribute to cell damage (Figure 1).

Proinflammatory cytokines, including interleukin-1 (IL-1), tumor necrosis factor-alpha (TNF-α), and interferon-gamma (IFN-γ), play pivotal roles in beta-cell destruction. These cytokines not only promote inflammation but also impair beta-cell function and trigger apoptosis. Chronic exposure of beta-cells to these inflammatory mediators is thought to induce a dysfunctional state termed ’beta-cell exhaustion’, diminishing insulin secretion and increasing susceptibility to apoptosis [10].

## 2. T1D Staging

**Figure 2:**
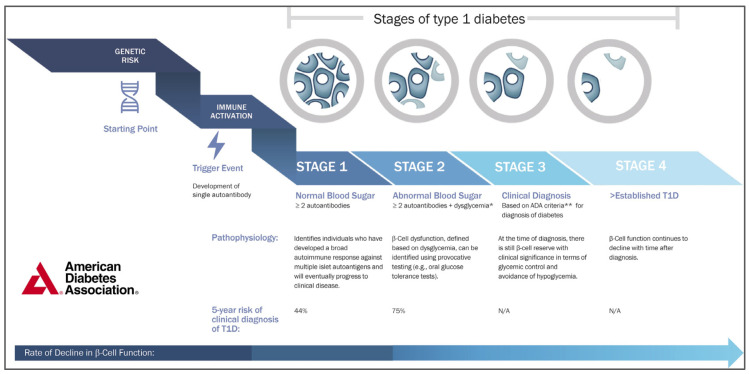
Definitions of stages of type 1 diabetes. * Dysglycemia defined according to the Consensus Guidance for Monitoring Individuals with Islet Autoantibody-Positive Pre-Stage 3 Type 1 Diabetes [11]. ** Because some patients are actually asymptomatic at the time that they cross the threshold for glucose-based criteria. From by permission [12].

T1D progresses through several stages, from genetic predisposition to overt clinical symptoms [1], and understanding these stages is key to early diagnosis and therapeutic intervention. The disease model is illustrated in Figure 2 and detailed below.

### 2.1. Pre-Stage 1: Genetic Predisposition and Immune Activation Phase

Certain environmental factors may lead to the early activation of autoreactive T cells in genetically predisposed individuals. These cells begin to target and destroy beta-cells even before the appearance of autoantibodies [13]. This early immune dysregulation is currently being studied for potential biomarkers that could predict the very earliest stages of autoimmunity before seroconversion [14].

### 2.2. Stage 1: Presymptomatic Autoimmunity

This stage is defined by the presence of two or more islet autoantibodies in normoglycemic individuals. Despite the underlying autoimmunity, insulin secretion remains sufficient to maintain euglycemia, and patients are asymptomatic. The most commonly detected autoantibodies include those targeting GAD65, insulin (IAA), insulinoma-associated antigen-2 (IA-2), and zinc transporter 8 (ZnT8A). Individuals at this stage are at a significant risk of progression [15,16].

### 2.3. Stage 2: Dysglycemia and Beta-Cell Dysfunction

In stage 2, individuals develop dysglycemia as beta-cell function declines, but full clinical symptoms of diabetes have not yet manifested. The recently published Consensus Guidance for Monitoring Individuals With Islet Autoantibody-Positive Pre-Stage 3 Type 1 Diabetes proposed for glycemic status staging at least two of the following, or meeting the same single criteria at two time points within 12 months: fasting plasma glucose (FPG) 100–125 mg/dL (5.6–6.9 mmol/L); 120 min oral glucose tolerance test (OGTT) 140–199 mg/dL (7.8–11.0 mmol/L); OGTT values > 200 mg/dL (>11.1 mmol/L) at 30, 60, and 90 min; HbA1c 5.7–6.4% (39–47 mmol/mol) or longitudinal > 10% increase in HbA1c from the first measurement with stage 2 T1D; and continuous glucose monitoring (CGM) values > 140 mg/dL (>7.8 mmol/L) for 10% of the time over 10 days’ continuous wear and confirmed by at least one other non-CGM glucose measurement test listed [11]. This stage marks subclinical T1D, in which insulin production is diminished but not yet critically deficient [1]. Stage 2 is a critical window for intervention, with efforts underway to prevent the progression to clinical diabetes using immunomodulatory therapies or beta-cell preservation strategies [17].

### 2.4. Stage 3: Symptomatic T1D

Stage 3 marks the clinical onset of T1D, which is characterized by symptomatic hyperglycemia (e.g., polyuria, polydipsia, and weight loss) due to severe insulin deficiency. The diagnostic criteria include one or more of the following: One random venous glucose >200 mg/dL (>11.1 mmol/L) with overt symptoms, 120 min OGTT > 200 mg/dL (>11.1 mmol/L), two random venous glucose > 200 mg/dL (>11.1 mmol/L), FPG > 126 mg/dL (> 7.0 mmol/L), laboratory-tested HbA1c > 6.5% (> 48 mmol/mol), and CGM values > 140 mg/dL (> 7.8 mmol/L) for 20% of time over 10 days’ continuous wear and confirmed by at least one other non-CGM glucose measurement test [11]. Most individuals at this stage have already lost much of their functional beta-cell mass. At this point, immediate insulin therapy is required to manage glucose levels and prevent acute complications, such as diabetic ketoacidosis (DKA).

### 2.5. Stage 4: Chronic Management and Complications

After diagnosis, individuals with T1D enter a chronic management phase, where the primary goal is to maintain glycemic control through exogenous insulin administration and CGM [18]. Intensive insulin therapy, including multiple daily injections or insulin pump therapy, is essential to prevent acute and long- term complications. Long-term glycemic variability increases the risk of microvascular (retinopathy, nephropathy, and neuropathy) and macrovascular complications (cardiovascular disease). Recent advances in diabetes technology, including hybrid closed-loop insulin delivery systems, have improved glycemic control and quality of life in patients [19]. Advances in immunotherapy and islet transplantation hold promise as potential avenues for delaying or reversing the course of the disease [20,21].

## 3. T1D Risk Prediction

T1D has a clear heritable risk component, which is largely explained by genetic variants in class I and II MHC genes, most notably the HLA haplotypes DR3-DQ2 and DR4-DQ8 [6]. Apart from the MHC locus, T1D is highly polygenic with > 90 associated single nucleotide polymorphisms (SNPs) including *INS* and *PTNP22*, which can be used to construct genetic risk scores (GRS) that predict the development of T1D [22]. Genetic screening can be conducted at birth to identify those predisposed to T1D, allowing for closer monitoring throughout childhood [23].

In addition to genetic factors, immunological markers also play a crucial role in risk prediction. Research on family cohorts and high-risk populations (such as those with first-degree relatives with T1D or carriers of high-risk HLA haploid genotypes) has allowed earlier identification and monitoring of individuals in stage 1 [24]. This presymptomatic phase represents a critical window for intervention, where risk prediction tools play a pivotal role. For example, studies involving the presence of autoantibodies, such as GADA and IAA, have shown that individuals with multiple autoantibodies are at a much higher risk of developing overt T1D [16]. These markers can often be detected years before the clinical onset of the disease, allowing for early intervention and better preparedness for disease management [24].

The progression from one to multiple autoantibodies is associated with a near certainty of progressing to clinical T1D, typically within a few years [16]. Therefore, screening for islet autoantibodies in individuals with genetic susceptibility can help to identify those in the early stages of the disease [25]. Risk prediction models enable healthcare providers to tailor treatments based on an individual’s unique risk profile. For instance, individuals identified as being at high risk for rapid disease progression may benefit from early initiation of insulin therapy or participation in clinical trials for disease-modifying therapies, such as immunotherapies aimed at preserving beta-cell function [26].

## 4. T1D Monitoring in Preclinical Stages

Blood glucose levels are the leading metabolic marker that provides insights into the progression of T1D. Impaired glucose tolerance, as measured by OGTT, often precedes the clinical diagnosis of T1D [27].

Recently, CGM has emerged as a promising tool for tracking the progression of T1D and guiding treatment decisions. Ongoing research is assessing CGM’s role in identifying individuals, including those with normal oral glucose tolerance, who are likely to progress rapidly to stage 3 T1D [11]. Professional CGM, which is blinded to the user, can help reduce the anxiety associated with fluctuating CGM readings and alarm notifications.

C-peptide measurements assess residual beta-cell function and serve as the primary biomarker in research settings for evaluating insulin production. They help differentiate between T1D, its stages, and type 2 diabetes [11].

## 5. T1D Arrest Attempts: Clinical Trials Update

By identifying individuals at high risk of developing T1D, early intervention strategies can be employed to delay or even prevent the onset of the disease while enabling more personalized treatment approaches for those who progress to clinical diagnosis. In recent years, significant research has been performed to halt the autoimmune attack on beta-cells in individuals at risk for or newly diagnosed with T1D. Notably, the U.S. Food and Drug Administration’s (FDA) recent approval of teplizumab, an anti-CD3 monoclonal antibody, for delaying the onset of stage 3 T1D in at-risk individuals represents a landmark achievement [28]. This approval was based on the Teplizumab Prevention Study, which included individuals with multiple autoantibodies and impaired glucose tolerance, but without clinical T1D [4]. Teplizumab acts by modulating immune responses, primarily targeting CD8+ T cells to deplete effector cells while promoting regulatory T cell populations. It also induces changes that lead to T cell exhaustion and the formation of gut-tropic regulatory cells, potentially moderating autoimmune attacks on pancreatic beta cells [29]. The results of another randomized trial with teplizumab in stage 3 have recently been published, showing benefits in the maintenance of C-peptide levels and lower insulin requirements at the 18-month follow-up [30]. Numerous clinical trials are exploring a diverse array of therapeutic modalities, ranging from antigen-specific therapies and immune modulators to innovative cell therapies, for disease prevention but also to preserve endogenous insulin function in individuals with newly diagnosed T1D (see Table 1 for a detailed description).

In addition to pharmacological interventions, lifestyle modifications, including dietary changes and physical activity, may play a role in delaying disease progression in individuals at high risk for T1D. For example, recent studies have suggested that a healthy, anti-inflammatory diet may reduce the risk of T1D in genetically predisposed individuals, although more research is needed to confirm these findings [62,63].

## 6. T1D Screening Programs Update

Most studies in this area have concentrated on first-degree relatives of individuals with T1D, despite this group of subjects representing only 10% of new cases. To enhance effectiveness, screening should be broadened to include the general population exhibiting an active immune response (indicated by the presence of islet-specific autoantibodies). This strategy has been initiated in Italy, where authorities recently approved T1D screening for all children in conjunction with celiac disease testing [64]. Similar initiatives have been adopted by other nations, including Germany, the United States, Australia, Israel, and the United Kingdom (Table 2).

### 6.1. Targeted Population: Relatives vs. General Population

Screening initiatives aimed at identifying individuals at risk for T1D have primarily focused on relatives of those affected by the disease to enhance efficiency and practicality. Individuals with a family member who has T1D face a 15 times higher risk of developing the condition compared to those without such a familial connection [65]. The lifetime risk of T1D for siblings of patients is approximately 6–7%, while for children of mothers and fathers with T1D, it ranges from 1.3–4% and 6–9%, respectively. These figures contrast with the 0.4% risk observed in the general population [6]. Consequently, about 90% of T1D cases occur in individuals without a family history of the disease. Recent advancements in therapies that can alter the progression of early-stage T1D have sparked discussions about the necessity and feasibility of implementing population-wide screening to detect those with elevated risk.

The Fr1da study conducted a pilot study for general population screening, examining islet autoantibodies in approximately 150,000 children aged two to five in Germany. Findings from Fr1da indicate that the likelihood of progressing from early-stage T1D to clinical T1D is comparable between the general population and those with a genetic predisposition to T1D [66]. Consistent with other research, a milder clinical presentation was observed at the onset of stage 3 T1D in children with an early-stage diagnosis who participated in education and monitoring programs [67]. The results of Fr1da, and other screening programs based on primary care, provide evidence supporting the practicality and effectiveness of widespread autoantibody screening in the population. Recently, the European Society of Pediatric Endocrinology (ESPE) has approved a Position Statement on Screening for T1D in the general population, expressing optimism that future advancements will overcome current challenges [68].

### 6.2. Genetic and/or Autoantibody (AA) Screening

Current population screening initiatives utilize genetic or autoantibody tests, which have provided valuable insights into disease progression and informed the timing of clinical screenings.

**Figure 3:**
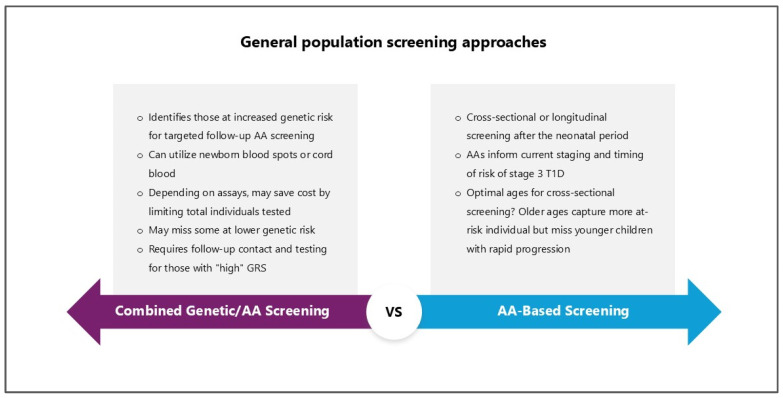
Combined genetic/AA-based screening versus an AA-based approach. Adapted with permission from [12].

AA: Tests demonstrate sufficient sensitivity and specificity to differentiate individuals with T1D from those without diabetes [69]. Several initiatives employ AAs for initial screening in children beyond the neonatal stage, including ASK (Autoimmunity Screening for Kids, Colorado) [70], T1Detect (U.S.), Early Detection of Type 1 Diabetes (Fr1da) [66], and Early Detection of Type 1 diabetes and Hypercholesterolemia in Lower Saxony (Fr1dolin) (Germany) [71]. While AA screening without prior genetic testing is costlier, it offers greater disease specificity (Figure 3). To screen for stages 1 and 2 T1D, all relevant autoantibodies would need to be examined using a small blood sample.

Genetics (HLA/GRS): The TEDDY (The Environmental Determinants of Diabetes in the Young) study employed HLA screening to collect data from over 8000 newborns, with the majority (90%) lacking a known family history of T1D [72]. Since 1994, the Type 1 Diabetes Prediction and Prevention Study (DIPP) has been conducted in three Finnish university hospitals, screening more than 250,000 infants [73]. This program examined cord blood samples from all newborns in these hospitals (25% of the national birth cohort) for HLA-conferred T1D susceptibility. Nearly 10% of the screened infants possessed such HLA genotypes and were invited to participate in follow-up until the age of 15 or T1D diagnosis. Launched in 2018 in Helsinki, Finland, the BABYSCREEN study (Newborn Screening for Genetic Susceptibility to Type 1 Diabetes and Celiac Disease and Prospective Follow-up Study) analyzed cord blood cells for HLA alleles associated with high T1D and celiac disease risk. Children carrying risk-associated haplotypes for either condition were invited to undergo AA testing at ages 1, 2, and 3. Of the 9000 children screened, 6.0% were identified to have a high genetic risk for T1D, 15.0% for celiac disease, and 4.1% for both diseases. The Global Platform for the Prevention of Autoimmune Diabetes (GPPAD) examines newborn blood spots from cord blood or primary care provider visits and calculates the GRS to identify those with a 10% risk of multiple AAs by age 6. Individuals with elevated genetic risk were enrolled in a primary prevention study. As of August 2022, GPPAD has screened over 350,000 newborns, with 1.1% showing an increased genetic risk [74]. In the United States, three new initiatives, CASCADE (https://cascadekids.org, accessed on 24 October 2024), Sanford PLEDGE (https://research.sanfordhealth.org/fields-of-research/diabetes/pledge, accessed on 24 October 2024), and PRiMeD [75], employ GRS obtained from dried blood spots or saliva samples. Individuals identified with “positive” GRS are then offered AA detection. The PLEDGE study specifically conducts AA testing during 2-year and pre-kindergarten check-ups, with a focus on incorporating these procedures into standard pediatric care practices and electronic health record systems.

### 6.3. Benefits of Screening Programs

T1D can present with preventable severe complications (DKA). Furthermore, the disease has negative effects on morbi-mortality and quality of life of patients and their families. Thus, there is a pressing need to create and implement effective strategies to identify individuals who could benefit from early intervention, regardless of whether they have a family member with T1D. Screening provides access to medical expertise, close monitoring, and educational programs. This strategy can dramatically lower the incidence of DKA at clinical diagnosis, reducing it from 25–62% to 4–6% [70]. Pre-clinical monitoring may also improve HbA1c levels at stage 3 diagnosis and lower complication risks [66,67,76]. Early identification of T1D in children could potentially mitigate the decline in metabolic function, which would ultimately reduce long-term complications such as brain and vascular damage linked to hyperglycemia and hypoglycemia [77]. Moreover, early T1D screening could become cost-effective owing to the prevention of DKA hospitalization and the anticipated reduction in diabetes complications [78]. Table 3 summarizes the benefits of early screening.

### 6.4. Follow-Up and Support Post-Screening

As described in Table 2, several studies have explored how to translate the management after screening for T1D into clinical practice. For instance, a qualitative study conducted in the UK explored parental perspectives on pediatric T1D screening and highlighted the importance of clear communication to encourage participation as well as the need for psychological support post-screening [79]. In Italy, the implementation of population screening for T1D via autoantibody detection has been accompanied by the development of a follow-up program for at-risk children, which includes recommendations for CGM and collaboration with healthcare providers to ensure comprehensive care [64]. Moreover, artificial intelligence has been proposed as a tool to enhance screening efficiency, offering personalized monitoring plans and operational feasibility [80]. Lastly, the Type 1 Diabetes Mellitus Screening Acceptability Scale has been developed to assess public attitudes toward screening, revealing strong psychometric properties and helping to guide culturally sensitive screening programs [81].

## 7. Spanish Health System, Strengths and Weaknesses for a Presymptomatic T1D Screening and Management Program

The costs of screening for presymptomatic beta-cell autoimmunity may vary across countries and depend on the particularities of each healthcare delivery system. The implementation of a T1D screening and management program requires a robust health system infrastructure, multidisciplinary cooperation, and targeted resource allocation. The Spanish public health system (SNS) is characterized by advantaged universal coverage and accessible primary care. However, it also faces challenges that need to be addressed to ensure the successful implementation of such a screening program. We describe the strengths and weaknesses of the SNS in relation to a T1D screening program, focusing on the aspects of feasibility, cost-effectiveness, and sustainability, as well as barriers to equitable access and engagement.

### 7.1. Universal Health Coverage and Accessibility

SNS provides universal healthcare, guaranteeing access to all citizens regardless of socioeconomic status. Spain’s robust primary care (PC) network serves as a backbone for public health initiatives. This is particularly advantageous for the large-scale implementation of a T1D screening program. A broad population can be reached, and therefore, reduces the potential disparities in access to screening. The comprehensive coverage of SNS includes primary care services, which are pivotal in presymptomatic screening programs. Additionally, the geographical distribution of healthcare centers, including in rural areas, helps to ensure that a screening program can reach a wide population.

### 7.2. The Spanish PC Pediatrician Staff

PC pediatricians are officially recognized as a standard professional resource in every healthcare area in Spain. This ensures that screening and follow-up care can be initiated in a timely manner. They can establish close relationships with patients, allowing for personalized communication strategies regarding the risks of T1D, which is crucial to engage patients and their families in a presymptomatic screening program.

### 7.3. Access to Specialized Care

While Spain has a universal health system, the decentralization of healthcare to its autonomous regions can lead to variability of healthcare services. It particularly applies to access to specialized endocrinology care and advanced diagnostic tools. This poses a challenge to ensure the uniform implementation of a T1D monitoring and follow-up program across the country, particularly in under-resourced or rural regions where access to pediatric endocrinologists and specialized diabetes care may be limited. The access to specialized care, such as immunotherapies, is uneven across the country. Specialized endocrinology services are often concentrated in larger cities, meaning that individuals in rural or underserved areas may face delays in receiving the necessary post-screening care. Given the importance of timely intervention in preventing the onset of T1D, disparities in access to specialists may undermine the efficacy of presymptomatic screening programs.

### 7.4. Experience with Other Preventive Programs

Spain’s newborn screening program, which tests for various metabolic and genetic disorders at birth, already has the laboratory, data management, and reporting systems necessary for population-based screening initiatives. Leveraging this infrastructure could allow for the integration of T1D screening, which could be performed alongside existing tests for metabolic disorders. The existing follow-up protocols in newborn screening programs—such as contacting families, scheduling confirmatory tests, and arranging care pathways—could be adapted to ensure continuity of care for those at risk of developing T1D. Additionally, vaccination programs utilize electronic health records (EHR) systems to track vaccination status, schedule reminders, and provide follow-up care. These digital systems could be adapted to track autoantibody results, coordinate follow-up testing, and monitor individuals identified as high-risk.

### 7.5. Research and Development Infrastructures

Spain is actively involved in biomedical research, including diabetes and immunological studies, which could support the introduction of screening programs. Collaborative networks among research institutes and hospitals could facilitate clinical trials, refinement of screening modalities, and integration of new immunotherapies into care protocols for at-risk individuals. Nevertheless, there are no current national guidelines that provide recommendations on optimal screening assays, frequency of testing based on age/risk, how to communicate the results to patients/caregivers, blood glucose monitoring protocols, and treatment guidance for those who need it.

### 7.6. Resources for Early Intervention Programs

Although the Spanish health system is competent in managing established diabetes, resource constraints may limit its capacity to effectively implement large-scale presymptomatic screening and early intervention programs. The financial demands associated with routine screening could strain the system.

### 7.7. Engagement and Participation of At-Risk Populations

Engaging individuals without a family history of T1D may present a significant barrier to widespread participation in a screening program. The absence of previous research studies in Spain limits the general population’s and healthcare administration’s awareness of the current possibilities for detecting and delaying the T1D process. This will require carefully designed communication strategies. Parental and healthcare provider perceptions of the benefits of screening should be considered for program success.

## 8. Conclusions and Call to Action

Despite remarkable improvements in our knowledge of the natural history of T1D progression and its management, it remains a challenging condition. The advent of teplizumab and other future immunomodulatory agents opens up promising possibilities for early preventive care. However, these must be weighed carefully against the costs, potential benefits, and broader impacts on patients and health systems.

The Spanish healthcare system has several strengths that position it well for the implementation of a presymptomatic T1D screening and management program, including universal health coverage, robust PC infrastructure, and experience in managing chronic diseases. However, significant challenges remain, particularly in terms of resource allocation, engagement of low-risk populations, and equitable access to specialized care across regions. The geographic specificity may limit the generalizability of our conclusions to other countries with different healthcare systems, populations, and resources. The decision to implement population screening programs should be based on robust clinical evidence and ethical considerations, aligning with broader public health goals. Spain and other countries should develop evidence-based guidelines with consistent recommendations to clinicians, accompanied by a follow-up program for at-risk subjects and their families. It is crucial to acknowledge and incorporate the perspectives of patients and caregivers in the decision-making process in healthcare settings. Future research should focus on comparative analyses across different healthcare systems, the long-term outcomes of specific therapies, and further evaluate the economic impact of screening programs.

## Figures and Tables

**Table 1:** Clinical studies evaluating T1D disease-modifying therapies.

Study	Design	Population	Intervention	Primary Outcome	Findings
Antigen-specific immune therapy (for tolerance induction)					
Skyler et al., 2002 (DPT-1) [31]	Multicenter, double-blind trial	Aab+ first-degree relatives with high-risk features (*n* = 339)	0.25 U/kg ultralente + annual 4-day continuous insulin infusion vs. no intervention	Time to diabetes	Follow-up: 3.7 years Insulin at the dosage used did not delay or prevent T1D
Skyler et al., 2005 (DPT-1) [32]	Multicenter, double-blind trial	Aab+ first-degree relatives with high-risk features (*n* = 372)	Oral insulin (7.5 mg/day) vs. placebo	Time to diabetes	Follow-up: 4.3 years Oral insulin did not delay or prevent T1D *
Näntö-Salonen et al., 2008 [33]	Multicenter, double-blind trial	Infants with high-risk HLA genotype and their siblings with high-risk HLA and multiple Aab+ (*n* = 264)	Intranasal daily recombinant human short-acting insulin vs. placebo	Time to diabetes	Follow-up: 1.7–2.0 years Intranasal insulin did not delay or prevent the development of T1D
Vandemeulebroucke et al., 2009 [33]	Multicenter, double-blind trial	IA-2A+ relatives (*n* = 50)	Parenteral regular human insulin twice a day	Time to diabetes	Follow-up: 47–52 months No difference in diabetes-free survival between the two groups
Bonifacio et al., 2015 (Pre-POINT) [34]	Unicenter, double-blind trial	Aab- children with a family history of T1D high-risk HLA haplotypes (*n* = 25)	7.5 mg to 67.5 mg of oral insulin or placebo	Antibody or T cell response to insulin	Follow-up: 12 months Daily oral administration of insulin resulted in an immune response
Krischer et al., 2017 [35] (TN07)	Multicenter, double-blind trial	Multiple Aab+ relatives with insulin Aab+ (*n* = 560)	7.5 mg daily oral recombinant human insulin vs. placebo	Time to diabetes	Follow-up: 2.7 years Oral insulin did not delay or prevent the development of T1D
EldingLarsson et al., 2018 (DiAPREV-IT) [36]	Double-blind trial	Multiple Aab+ children with GADA+	2 injections of 20 μg GAD-Alum or placebo, 30 days apart	Safety and cumulative incidence of diabetes	Follow-up: 4.92 years GAD-Alum did not affect progression to T1D
Assfalg et al., 2021 (Pre-POINT early) [37]	Unicenter, double-blind trial	Aab- children with a family history of T1D high-risk HLA haplotypes (*n* = 44)	7.5 mg to 67.5 mg of oral insulin or placebo	Antibody or T cell response to insulin	Follow-up: 12 months No differences in immune responses to insulin
Lugvinsson et al., 2021 [38]	Multicenter, double-blind trial	Children and young adults with recently diagnosed T1D and GADA+ carrying HLA DR3-DQ2 (*n* = 48)	3 intralymphatic injections (1 month apart) with 4 μg GAD-alum and oral vitamin D or placebo	Endogenous insulin production	Follow-up: 15 months The combination improved stimulated C-peptide levels
Lugvinsson et al., 2022 [39]	Multicenter, double-blind trial	Children and young adults with recently diagnosed T1D and GADA+ carrying HLA DR3-DQ2 (*n* = 330)	3 intralymphatic injections of rhGAD65 and oral vitamin D or placebo	Endogenous insulin production and glycemic control	Follow-up: 22 months Ongoing
PINIT Study (NCT03182322)	Multicenter, double-blind trial	Aab- children with the HLA DR3/4-DQ8 genotype or with a first degree relative with T1D and at least one high-risk HLA haplotype	Intranasal insulin or placebo	Antibody or T cell response to insulin at any time point during treatment	Completed; pending results
Fr1da Insulin Intervention Study (NCT02620072)	Multicenter, double-blind trial	Children with multiple Aab	7.5 mg to 67.5 mg of oral insulin or placebo	Time to dysglycemia or diabetes	Ongoing
Immune modulation (for restoring the balance)					
Gale et al., 2004 (ENDIT) [40]	Multicenter, double-blind trial	Aab+ relatives (*n* = 552)	Oral nicotinamide (1.2 g/m^2^) or placebo	Development of diabetes	Follow-up: 5 years No differences in T1D incidence
Mastraendrea et al., 2009 [41]	Unicenter, double-blind trial	Children and adolescents with newly diagnosed T1D (*n* = 18)	Etanercept or placebo	HbA1C	Follow-up: 24 weeks HbA1C values were lower in the etanercept group
Pescovitz et al., 2009 [42]	Multicenter, double-blind trial	Children and adults with newly diagnosed T1D (*n* = 87)	Rituximab or placebo	Preservation of beta-cell function	Follow-up: 24 weeks Rituximab improved stimulated C-peptide levels
Orban et al., 2011 [43]	Multicenter, double-blind trial	Children and adults with newly diagnosed T1D (*n* = 112)	Abatacept or placebo	Preservation of beta-cell function	Follow-up: 1 year Abatacept improved stimulated C-peptide levels
Ambery et al., 2014 [44]	Multicenter, double-blind trial	Adolescents with newly diagnosed T1D (*n* = 54)	(Low-dose) otelixizumab or placebo	Endogenous insulin production	Follow-up: 12 months No improvement in stimulated C-peptide levels
Aronson et al., 2014 [45]	Multicenter, double-blind trial	Individuals with newly diagnosed T1D (*n* = 218)	(Low-dose) otelixizumab or placebo	Endogenous insulin production	Follow-up: 12 months No improvement in stimulated C-peptide levels
Rigby et al., 2015 [46]	Multicenter, double-blind trial	Individuals with newly diagnosed T1D (*n* = 49)	Alefacept or placebo	Endogenous insulin production	Follow-up: 15 months after last dose Alefacept improved stimulated C-peptide levels
Haller et al., 2019 [47]	Multicenter, double-blind trial	Individuals with newly diagnosed T1D (*n* = 89)	Low-dose ATG ± GCSF or placebo	Endogenous insulin production	Follow-up: 2 years Only low-dose ATG improved stimulated C-peptide levels
Herold et al., 2019 [4]	Multicenter, double-blind trial	Aab+ relatives with high-risk features (*n* = 76)	Teplizumab or placebo	Time to diabetes	Follow-up: more than 3 years Teplizumab delayed progression to clinical T1D
Quattrin et al., 2020 (T1GER) [48]	Multicenter, double-blind trial	Children and young adults with newly diagnosed T1D (stage 3) (*n* = 84)	Golimumab or placebo	Endogenous insulin production	Follow-up: 52 weeks Golimumab improved stimulated C-peptide levels
Keymeulen et al., 2021 [45]	Multicenter, double-blind trial	Individuals with newly diagnosed T1D (*n* = 30)	Otelixizumab or placebo	Endogenous insulin production	Follow-up: 24 months Otelixizumab 9 mg improved stimulated C-peptide levels
Von Herrath et al., 2021 [49]	Multicenter, double-blind trial	Adults with recently diagnosed T1D (*n* = 308)	Anti-IL-21 plus liraglutide or placebo	Endogenous insulin production	Follow-up: 54 weeks The combination improved stimulated C-peptide levels
Libman et al., 2023 [50]	Multicenter, double-blind trial	Individuals with stage 1 T1D	Hydroxychloroquine or placebo	Progression to stage 2 T1D	Follow-up: 23.3 months Prematurely stopped due to futility
Russell et al. 2023 [51]	Multicenter, double-blind trial	Aab+ relatives (*n* = 212)	Abatacept or placebo	Time to glucose intolerance or diabetes	Follow-up: 36.9 months No significant delay in progression to glucose intolerance
Ramos et al., 2023 [30]	Multicenter, double-blind trial	Children with newly diagnosed T1D (stage 3) (*n* = 328)	Teplizumab or placebo	Preservation of beta-cell function	Follow-up: 78 weeks Teplizumab improved stimulated C-peptide levels
Mathieu et al., 2024 [52]	Multicenter, double-blind trial	Adolescents and adults with recently diagnosed T1D (stage 3) (*n* = 328)	Oral AG019 ± teplizumab	Metabolic and immune endpoints	Follow-up: 12 months AG019/teplizumab stabilized or improved metabolic variables
Tatovik et al., 2024 [53]	Multicenter, double-blind trial	Adolescents with new-onset T1D (*n* = 72)	Ustekinumab or placebo	Endogenous beta-cell function	Follow-up 12 months Ustekinumab improved stimulated C-peptide levels
Perdersen et al., 2024 [54]	Multicenter, double-blind trial	Children and adolescents with recent-onset T1D	Inactivated quadrivalent influenza vaccine or placebo	Endogenous beta-cell function	Ongoing
NCT04524949 (IMPACT study)	Multicenter, double-blind trial	Adults with recently diagnosed T1D	IMCY-0098 or placebo	Endogenous beta-cell function	Ongoing
Harnessing the beta-cell loss					
Ovalle et al., 2018 [55]	Multicenter, double-blind trial	Adults with recent-onset clinical T1D (*n* = 32)	Verapamil or placebo	Endogenous beta-cell function	Follow-up: 12 months Verapamil improved stimulated C-peptide levels
Gitelman et al., 2021 [56]	Multicenter, double-blind trial	Adults with recent-onset clinical T1D (*n* = 67)	Imatinib or placebo	Endogenous beta-cell function	Follow-up: 12 months Imatinib improved stimulated C-peptide levels
Forlenza et al., 2023 [57]	Multicenter, double-blind trial	Children and adolescents with newly diagnosed T1D (*n* = 88)	Verapamil or placebo	Endogenous beta-cell function	Follow-up: 52 weeks Verapamil improved stimulated C-peptide levels
Krogvold et al., 2023 [58]	Multicenter, double-blind trial	Children and adolescents with newly diagnosed T1D (*n* = 96)	Pleconaril and ribavirin or placebo	Endogenous beta-cell function	Follow-up: 12 months The combination improved stimulated C-peptide levels
Waibel et al., 2023 [59]	Multicenter, double-blind trial	Children and young adults with newly diagnosed T1D (*n* = 91)	Baricitinib or placebo	Endogenous beta-cell function	Follow-up: 48 weeks Baricitinib improved stimulated C-peptide levels
Cell therapy					
Ramzy et al., 2021 [60]	Phase 1/2 study	Adults with established T1D (*n* = 15)	Subcutaneously implanted PEC	Safety and efficacy parameters	Follow-up: 1 year Reduced insulin requirement
Leão et al., 2024 [61]	Retrospective cohorts	Patients with recent-onset T1D	Infusion of ASC + vitamin D	Partial clinical remission	Follow-up: 36 months Less insulin requirement than controls

* Post hoc analysis showed delay in progression to T1D in relatives with insulin Aab ≥ 80 U/mL. Aab, autoantibody; AG019, food-grade *Lactococcus lactis* bacteria genetically modified to express human proinsulin and human IL-10; ASC, adipose tissue stromal/stem cell; ATG, anti-thymocyte globulin; T1D, type 1 diabetes; GCSF, granulocyte colony-stimulating factor; HbA1C, glycated hemoglobin; HLA, human leukocyte antigen; Aab+, autoantibody-positive; GADA, glutamic acid decarboxylase autoantibody; PEC, pancreatic endoderm cells; rhGAD65, recombinant human glutamic acid decarboxylase 65 kDa.

**Table 2:** Screening programs.

Program	Target Population	Location	Number Screened	Method	Positivity Rates	Remarks
Screening programs for relatives of patients with T1D						
TrialNet Pathway to Prevention (TN01)	Relatives aged 3–45 years	U.S., Canada, Europe, Australia	>250,000	RBA: IAA and GADA, followed by IA-2A, ZnT8A, and ICA if positive	AA+: 5% ≥2 AA+: 2.5%	Main aim: identify participants eligible for clinical trials
INNODIA	Relatives and general population	Europe	>4400	RBA	1 AA+: 6.0% ≥2 AA+: 2.6% >2 AA+: 1.0% 3 AA+: 0.9% 4 AA+: 0.8%	
Bart’s Oxford (BOX) Family Study	Relatives	United Kingdom	6000	RBA: IAA, GADA, IA-2A, ZnT8A	1 AA+: 6% ≥2 AA+: 2%	Family members recruited at diagnosis of a proband (<21 years old) in the study area
Type1Screen	Relatives aged 2–30 years	Australia and New Zealand	>700	IAA: RBA or ADAP; GADA, IA-2A, ZNT8A, ELISA, or ADAP	AA+: 5% 1 AA+: 1.9% ≥2 AA+: 3.9%	Family members recruited by health professionals, emails, and social media
Screening programs for general population with genetic risk						
DIPP	Age 0.25–15 years with high-risk HLA genotypes	Finland	>250,000	HLA genotyping followed by RBA: IAA, GADA, IA-2A, ZnT8A	∼10% of screens with high-risk HLA ≥ 2 AA+: by 2 years: 2.2% by 5 years: 3.5% by 15 years: 5.0%	Follow-up for AA screening at 3- to 12-month intervals up to age 15 years
BABY- SCREEN	Newborns to 3 years with high-risk HLA for T1D and/or celiac disease	Helsinki, Finland	Target for HLA screening: 30,000; > 9000 tested	HLA genotyping followed by RBA: IAA, GADA, IA-2A, ZnT8A, tTGA	By 1 year: 1 AA+: 5.3% ≥2 AA+: 1.8% By 2 years: 1 AA+: 6.5% ≥2 AA+: 3.7%	Newborn infants from the general population were screened at birth for HLA-conferred susceptibility to T1D and celiac disease
GPPAD	Infants < 1 month of age	Germany, U.K., Poland, Belgium, and Sweden	>275,000 (1.72% first-degree relatives)	47-SNP GRS to identify those with > 10% risk of ≥ 2 AA+ by age 6 years	1.1% with increased genetic risk	At-risk infants are candidates for a primary prevention trial
PLEDGE	Age < 6 years	North and South Dakota and Minnesota, U.S.	Intended = 33,000	GRS, RBA	Pending results	GRS with newborn screen or study entry; AA testing at ∼2 and 5 years
CASCADE	Age ≥ 1 year	Northwest U.S.	Intended = 60,000	GRS, RBA: GADA, IAA, ZnT8A, tTGA; LIPS for IA-2A	Pending results	Initial GRS screen, at-risk infants followed for T1D and celiac disease
PRiMeD	Age 2–16 years	Virginia, U.S.	3818	82-SNP GRS, RBA: IAA, GADA, IA-2A, ZnT8A	542 (14.2%) with high GRS AA testing in progress	Low rate of AA testing due to the SARS-CoV-2 pandemic
Screening programs for general population based on AA testing						
Fr1da	Age 1.75–10.99 years	Germany	>150,000	ELISA: GADA, IA-2A, ZnT8A/LIPS: IAA; confirmation with RBA: IAA, GADA, IA-2A, ZnT8A	≥2 AA+: 0.3%	Follow-up for metabolic staging (OGTT)
Fr1dolin	Age 2–6 years	Germany	>15,000	ELISA: GADA, IA-2A, ZnT8A; confirmation with RBA: IAA, GADA, IA-2A, ZnT8A	≥2 AA+: 0.35%	Combined screening for T1D risk and familial hypercholesterolemia Follow-up for metabolic staging (OGTT)
T1Detect (JDRF)	Age ≥ 1 year	U.S.	Up to 2000/month	ADAP: GADA, IA-2A, IAA	Nonrelatives: 1 AA+: 12% ≥2 AA+: 5.4% Relatives: 1 AA+: 12% ≥2 AA+: 5.7%	Of the first 800 tests, 203 (25.4%) were from the general population
ASK	Age 1–17 years (currently, also adults)	Colorado, U.S.	25,738	RBA with ECL confirmation: IA-2A, GADA, IAA, ZnT8A, tTGA	AA+: 3.4% ≥2 AA+: 0.52% Single high-affinity AA+: 0.58%	Screening for T1D, celiac disease, and SARS-CoV-2 Ab 4.84% with first-degree relative with T1D
ELSA	Age 3–13	United Kingdom	20,000	ELISA: GADA, IA-2A, ZnT8A; confirmation with RBA: IAA, GADA, IA-2A, ZnT8A	Pending results	All AA+ children and their families are invited to an education session about the signs and symptoms of T1D and the risk of progression to stage 3.
T1DRA	Age 18–70	United Kingdom	20,000	ELISA: GADA, IA-2A, ZnT8A	Pending results	People at high risk will be offered information about the symptoms T1D and its management, along with continued monitoring
UNISCREEN	Age 1–100	Milan, Italy	1500	LIPS: GADA, IAA, IA-2A, ZnT8A	Pending results	Part of a universal screening for early detection of chronic autoimmune, metabolic and cardiovascular diseases
T1Early	Preschool age: 3.5–4 years	United Kingdom	N/A	LIPS: GADA, IA-2A, ZnT8A	Pending results	AA+ children will undergo metabolic staging
ADIR	Age 9–18 months and 5 years	Israel	Up to 50,000	ADAP: GADA, IA-2A, IAA	Pending results	AA+ children (stage 1 or 2 T1D) will be educated about the appearance of clinical signs of diabetes
JDRF Australia General Population Screening Pilot	Newborns, infants, and 2–10 years	Australia	3000 in each cohort	GRS, ADAP for IAA, GADA, IA-2A, ZNT8A	Pending results	Head-to-head comparison of autoantibody and genetic screening models
Birth cohorts (relatives and general population)						
BABYDIAB	Children of parents with T1D	Germany 1989–2000	2364	ICA and RBA: IAA, GADA, IA-2A, ZnT8A and TTG AA	AA+: 220 (9%) ≥ 2 AA+: 123 (5%)	From 3 years, yearly OGTT monitoring if AA+
DAISY	Newborn GP and relatives < 4 years	Colorado, U.S. 1993–2004	Newborns: 32,114	RBA and ECL: IAA, GADA, IA-2A, ZnT8A, tTGA	1424 GP newborns and 1123 relatives identified and followed AA+: 8% ≥2 AA+: 5%	Genetically at-risk newborns based on HLA genotyping and relatives followed at 9, 15, 24 months and annually thereafter until age 20 years AA+ followed until 30 years
DEW-IT	GP newborn	Washington, U.S. 1995–2001 2010–2012	42,000 blood spots tested	HLA genotyping; RBA: IAA, GADA, IA-2A, and later, ZnT8A	AA+: 173 (5%) ≥2 AA+: 170 (5%)	Consenting families received HLA genotyping of dried newborn blood spots followed by AA monitoring ofat-risk individuals
DiPiS	GP newborns	Sweden 2000–2004	35,688	HLA genotyping; RBA: IAA, GADA, IA-2A, ZnT8A	AA+: 184 (4%)≥2 AA+: 100 (2%)	Positive screens with yearly follow-up. Those with ≥ 2 AA+ followed every 3 months
TEDDY	Newborns in both relatives and GP	U.S., Finland, Germany, Sweden 2004–2010	424,788	HLA genotyping; RBA: IAA, GADA, IA-2A, tTGA	21,589 (0.05%) of screens with high-risk HLA; 8676 parents consented to follow-up	High-risk newborns followed every 3–6 months for 15 years for AAs and T1D, with documentation of potential environmental contributors. 90% without a known relative with T1D

AA, autoantibody; ADAP, agglutination-polymerase chain reaction; ELISA, enzyme-linked immunosorbent assays; GADA, glutamic acid decarboxylase autoantibody; GP, general population; GRS, genetic risk score; LIPS, luciferase immunoprecipitation systems; N/A, not available; OGTT, oral glucose tolerance test; RBA, radiobinding assays.

**Table 3:** Advantages of T1D early screening programs.

Potential Advantages
Access to and development of preventive therapies
Reduction in DKA
Reduction of symptoms, weight loss
Reduction in hospitalization (rate and days)
Improved beta-cell function
Improved quality of life, reduced psychological stress
Smooth transition to insulin therapy at the optimal time

## Data Availability

No new data were created or analyzed in this study. Data sharing is not applicable to this article.

## Appendix A

AGORA Diabetes collaborative group: Abreu Padin, Cristina; Aguilera Hurtado, Eva; Azriel Mira, Sharona; Barajas Galindo, David Emilio; Bartual Rodrigo, Amparo; Bellido Guerrero, Diego; Blanco Carrasco, Antonio Jesus; Botana Lopez, Manuel Antonio; Brito Sanfiel, Miguel Angel; Capel Flores, Ismael; Castaño Gonzalez, Luis; Chamorro Martin, Jose Luis; Codina Marcet, Mercedes; Cuesta Hernandez, Martin; Darias Garzon, Ricardo; Dominguez Riscart, Jesus; Duran Rodriguez-Hervada, Alejandra; Enes Romero, Patricia; Galvan Diaz, Beatriz; Gimeno Orna, Jose Antonio; Gomez Peralta, Fernando; Gonzalez Albarran, Maria Olga; Gonzalez Perez De Villar, Noemi; Jimenez Varas, Ines; Lechuga Sancho, Alfonso Maria; Lopez De La Torre Casares, Martin; Lopez-Guzman Guzman, Antonio Jesus; Marco Martinez, Amparo; Mesa Pineda, Alex; Monreal Villanueva, Marta Maria; Moreira Rodriguez, Manuela; Ortola Buigues, Ana; Peteiro Gonzalez, Diego; Picon Cesar, Maria Jose; Pines Corrales, Pedro Jose; Pomares Gomez, Francisco Jose; Pujante Alarcon, Pedro; Riaño Galan, Isolina; Roldan Martin, Maria Belen; Ros Perez, Purificación; Ruiz De Adana Navas, Maria Soledad; Santos Mazo, Ruth Estefania; Soto Gonzalez, Alfonso.
